# 3-OBA Is Not an Antagonist of GPR81

**DOI:** 10.3389/fphar.2021.803907

**Published:** 2022-01-03

**Authors:** Mohammad Ali Mohammad Nezhady, Sylvain Chemtob

**Affiliations:** ^1^ Programmes en Biologie Moléculaire, Faculté de Médecine, Université de Montréal, Montreal, QC, Canada; ^2^ Centre de Recherche du CHU Sainte-Justine, Montreal, QC, Canada

**Keywords:** GPR81, lactate, 3-hydroxy-butyrate acid, antagonist, signalling

HCAR1, commonly known as GPR81, is a G-Protein Coupled Receptor (GPCR) and has been deorphanized more than a decade ago. Lactate is the endogenous ligand of GPR81, and many high-potential pharmacological agonists have been developed for its activation. Although some reports mention using 3-hydroxy-butyrate acid (3-OBA) as an antagonist of GPR81 thus inferring GPR81-mediated signaling mechanisms for their observed effects, there is no evidence for such an antagonistic activity in 3-OBA against GPR81. In fact, to this date, there is no report for an antagonist or an inhibitor of GPR81 at all, whereas 3-OBA is a ligand for HCAR2 (GPR109A) (Blad et al., 2011).

In a recent paper, Chen et al. used 3-OBA as the antagonist of GPR81 in combination with metformin and PD-1/PD-L1 blockade to demonstrate enhanced antitumor efficacy of later compounds (Chen et al., 2021). Their whole hypothesis is based on inhibition of GPR81 signaling that would increase the efficacy of metformin and PD-1/PD-L1 inhibition. The only method they used is inhibition of GPR81 signaling by 3-OBA to test their hypothesis. They attributed all the observed effects such as cell growth, metabolism, and T cell activation to GPR81 signaling. All of their conclusions are scientifically unfounded as 3-OBA is not a proven antagonist of GPR81 and since they have not used any other experiments to validate the GPR81-mediated effects (e.g., RNAi, knockout/knockdown). In another recent paper by Yang et al. (2021), authors used 3-OBA as an antagonist for GPR81 to investigate the role of this receptor in lactate-induced HMGB1 acetylation. Initially, they show that lactate is able to promote HMGB1 acetylation. They also show that this acetylation is independent of the lactate acidity since there is a similar HMGB1 acetylation when cells are treated with sodium lactate. GPR81 as the main known receptor for lactate signaling is their first guess to induce HMGB1 acetylation, and to this end they used 3-OBA as an antagonist for GPR81. They observed that prior treatment of cells with this putative antagonist reduces lactate-mediated HMGB1 acetylation. Authors have used this assumption in a previous publication (Yang et al., 2020) as well and mistakenly draw conclusion that TNFα production upon lactate treatment in LPS-stimulated macrophages is mediated by GPR81 signaling. However, based on GPR81 knockdown used in their previous study, findings using 3-OBA are incongruent. Moreover, these authors do not provide a reference for their rationale on using 3-OBA as GPR81 antagonist in both papers. Importantly, the use of 3-OBA as an antagonist of GPR81 is not limited to these authors. Khatib-Massalha et al. also used 3-OBA to inhibit GPR81 and indicated its pharmacological inhibition decreases the effect of lactate on neutrophil mobilization from bone marrow (Khatib-Massalha et al., 2020). However, alongside their so-called pharmacological inhibition of GPR81, they used GPR81 knockout animals to further prove their points which keeps their conclusion intact. Lee et al. as well used 3-OBA as the inhibitor of GPR81 and suggested various factors are expressed through GPR81-mediated signaling which are important in promoting intestinal stem cell-mediated epithelial development (Lee et al., 2018). Although they too used gene knockout mice to ascertain their conclusion, findings applying to other experiments inconsistently relied on 3-OBA as an inhibitor. The latter two papers refer to Shen et al. for their use of 3-OBA as a GPR81 antagonist (Shen et al., 2015). But Shen et al. in turn refer to a review paper for their claim on 3-OBA being the antagonist of GPR81 (Blad et al., 2011). Importantly, it should be underlined that there is no suggestion in the entire review paper to indicate that 3-OBA inhibits GPR81 (HCAR1). As a matter of fact, the review paper clearly indicates that no antagonist is known for HCAR1 to date. Notably, it should be emphasized that the review paper points to signaling activity of a different HCAR, precisely HCAR2 (GPR109A) for which 3-OBA is an agonist.

As mentioned above, some of the minor effects seen by 3-OBA are consistent with GPR81 knockdown or knockout experiments; however, this does not establish 3-OBA as an antagonist of GPR81. 3-OBA could simply act as a modulator of GPR81. However, current experimental evidence does not support such an activity.

Altogether, there is no experimental proof that 3-OBA acts as an antagonist of GPR81; to date, we are not aware of a specific antagonist of GPR81. Accordingly, this compound should not be used to investigate GPR81-selective signaling. Otherwise, one can be misled by the role of HCAR1 (GPR81) versus that of HCAR2 (GPR109A) for which 3-OBA is an agonist ligand (Offermanns et al., 2011).
